# The Microbiota–Gut–Brain Axis: Psychoneuroimmunological Insights

**DOI:** 10.3390/nu15061496

**Published:** 2023-03-20

**Authors:** Giuseppe Marano, Marianna Mazza, Francesco Maria Lisci, Michele Ciliberto, Gianandrea Traversi, Georgios Demetrios Kotzalidis, Domenico De Berardis, Lucrezia Laterza, Gabriele Sani, Antonio Gasbarrini, Eleonora Gaetani

**Affiliations:** 1 Department of Geriatrics, Neuroscience and Orthopedics, Institute of Psychiatry and Psychology, Fondazione Policlinico Universitario “A. Gemelli” IRCCS, Università Cattolica del Sacro Cuore, 00168 Rome, Italy; 2 Unit of Medical Genetics, Department of Laboratory Medicine, Fatebenefratelli Isola Tiberina-Gemelli Isola, 00168 Rome, Italy; 3 Department of Neurosciences, Mental Health and Sensory Organs (NESMOS), Sant’Andrea Hospital, Sapienza University of Rome, 00189 Rome, Italy; 4 Department of Mental Health, ASL 4, 64100 Teramo, Italy; 5 CEMAD Digestive Diseases Center, Fondazione Policlinico Universitario “A. Gemelli” IRCCS, Università Cattolica del Sacro Cuore, 00168 Rome, Italy; 6 Internal Medicine and Gastroenterology, Fondazione Policlinico Universitario “A. Gemelli” IRCCS, 00168 Rome, Italy; 7 Department of Medical and Surgical Sciences, Fondazione Policlinico Universitario “A. Gemelli” IRCCS, Università Cattolica del Sacro Cuore, 00168 Rome, Italy

**Keywords:** gut microbiota, brain, autoimmunity, nutrients, mood

## Abstract

There is growing interest in the role that the intestinal microbiota and the related autoimmune processes may have in the genesis and presentation of some psychiatric diseases. An alteration in the communication of the microbiota–gut–brain axis, which constitutes a communicative model between the central nervous system (CNS) and the gastro-enteric tract, has been identified as one of the possible causes of some psychiatric diseases. The purpose of this narrative review is to describe evidence supporting a role of the gut microbiota in psychiatric diseases and the impact of diet on microbiota and mental health. Change in the composition of the gut microbiota could determine an increase in the permeability of the intestinal barrier, leading to a cytokine storm. This could trigger a systemic inflammatory activation and immune response: this series of events could have repercussions on the release of some neurotransmitters, altering the activity of the hypothalamic–pituitary–adrenal axis, and reducing the presence of trophic brain factors. Although gut microbiota and psychiatric disorders seem to be connected, more effort is needed to understand the potential causative mechanisms underlying the interactions between these systems.

## 1. Introduction

**Figure 1:**
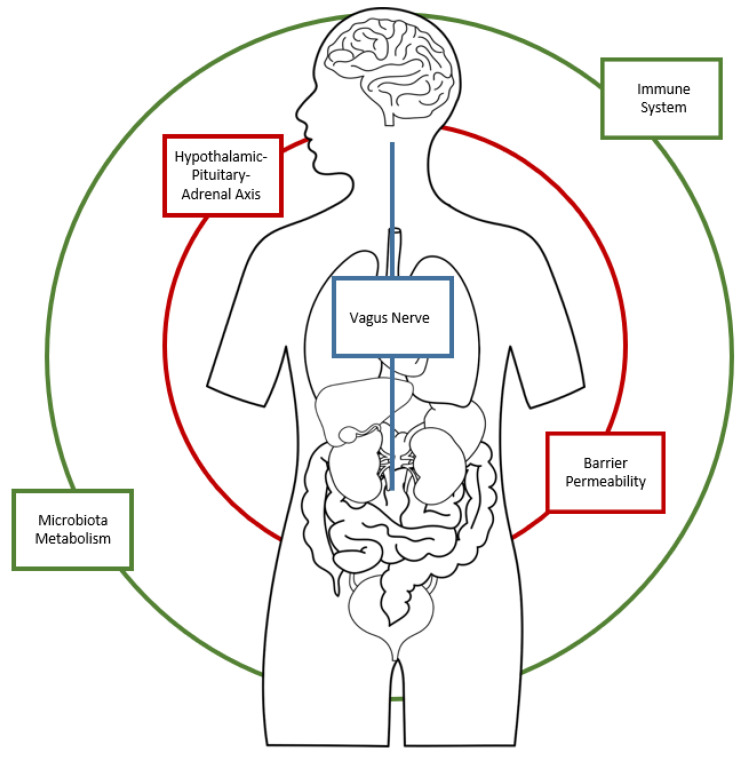
The Microbiota–Gut–Brain axis.

**Figure 2:**
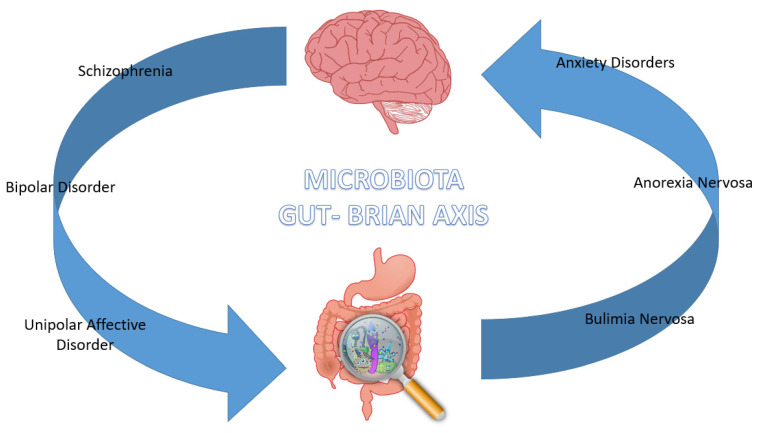
Microbiota–Gut–Brain axis involvement in Psychiatric Disorders.

Human microbiota is formed by various populations of symbiotic and commensal microorganisms, mainly formed by bacterial species, located in the gastrointestinal tract and mainly at the colon area. The colonization process begins at birth and continues throughout life, conditioned by genetic and epigenetic factors. The intestinal functions of gut microbiota are to facilitate digestive processes, favor nutrients’ metabolic processes and the synthesis of vitamin. In addition, intestinal flora plays important immune roles affecting metabolism and brain activity, creating a real gut–brain axis that puts in reciprocal communication these two apparatuses through the integration of the central, the peripherical and the autonomous nervous system [1,2]. The gut–brain axis communicates via neuronal message transmission carried by vagal affections, endocrine signals transported by intestinal hormones and immune signals conveyed by cytokines. Given the extensive neural network in the gastrointestinal area, ingested substances can trigger messages to the CNS related to macronutrients and caloric value through individualized and specific detection mechanisms situated throughout the gastrointestinal region [1]. Microbiota contributes to the homeostasis of the gut–brain axis, also mediating the immune processes and widening the field of action to a gut–brain immune system (Figure 1). This complex connection network between the immune and central nervous system is demonstrated by studies in rodents that highlighted how acute stress could increase the activity of nuclear pro-inflammatory factor kB (NF-κB), the activity of tumor necrosis factor intracellular-α (TNF-α) convertase, and levels of TNF-α, prostaglandin E2 (PGE2) and cyclooxygenase-2 (COX-2) in the prefrontal cortical region [2]. Alterations in the microbiota–gut–brain axis are not only linked to metabolic and immune disorders but also with psychiatric diseases, including schizophrenia, anxiety, mood disorders and neurodevelopmental disorders [3] (Figure 2).

### 1.1. The role of the Microbiota in the regulation of the Gut–Brain Axis

The intestinal flora is formed of 10^14 units of microbial cells that are represented by two dominant microbes, *Bacteroidetes* and *Firmicutes*, constituting 75–80% of the total. Phyla *Protobacteria*, *Acinetobacteria*, *Fusobacteria* and *Verrucomicrobia* represent a relatively lower proportion and interact with other intestinal microorganisms playing an important role in human health, interfacing with the immune and nervous system. In a state of health, they are in balance with the activity of the host cells, ensuring the physiological homeostasis of the gut–brain axis [4,5,6,7]. The microbiota takes an active part in the digestive processes by promoting the absorption of nutrients through the intestinal epithelial membranes. The integrity of the latter is guaranteed by the microbiota, which regulates the cellular metabolism and the development of the immune response [8]. The alteration of the regulation of the permeability of the blood–intestinal barrier can activate the processes of innate immunity, promoting the systemic and cerebral inflammatory state, and giving rise to susceptibility to stress and psychiatric pathologies. In fact, the commensal flora coordinates the downregulation processes of epithelial inflammatory responses, the expression of antimicrobial proteins, the defense of the epithelial surface through the production of mucus and the repair of damage to intestinal tissue [9]. Numerous factors, including age, geography [10], diet [11], drug use [12], toxins, infectious agents, and host genetics [12], can affect a healthy and properly functioning microbiota. In addition, the neonatal microbiota at birth is influenced by the maternal microbiota during childbirth and lactation [13,14]. According to recent studies, the gut microbiota could generate different types of neurotransmitters; for example, *Bifidobacterium* and *Lactobacillus* can produce γ-aminobutyric acid (GABA) [15], with enhancement of the inhibitory pathway in brain networks, whereas Lactobacillus and Oscillibacter increase gene expression of tryptophan synthase with incremented production of serotonin [16]. About 95% of 5-HT comes from this synthetic path. This production of neurotransmitters is affected by several elements, including vagus nerve stimulation and immune system functioning. Excitation of the afferent vagal pathways, via G-protein-coupled receptors [17] or histone deacetylases [18], results in modulation of monocytes, macrophages, neutrophils and dendritic cells functioning, also affecting the recruitment and differentiation of T cells [19]. An important role in the regulation of the gut–brain axis is played by the vagus nerve, thanks to its large network of nerve endings located within intestinal muscles at the submucosa and mucosa level (about 90% of total vagus nerve endings). This activity takes place in the inhibition of gastric vacuum and promotion of secretion of gastrointestinal glands, leading to the process of digestion and absorption of micro and macronutrients [20]. The vagus nerve activity goes well beyond the gut area, thus regulating memory, emotion and cognition due to the extended connections between the intestinal tract and cerebral cortex, amygdala and hippocampus [21]. The vagus nerve can directly perceive signals of neural activity from metabolites of intestinal flora through various pathways, including the afferent sensory pathway and a heterogeneous receptors’ network located on the surface of the vagus nerve (i.e., 5-HT and dopamine receptors, Toll-like receptors (TLR) 4, and free fatty acid receptors) [22]. Changes on this signaling architecture could lead to functional changes, as demonstrated by neuroimaging studies, which could contribute to the development of mental disorders such as substance dependence [23], mood disorders and eating disorders [23,24].

### 1.2. The Role of Autoimmunity

From the earliest stages of life, the gut microbiota plays a central role in the regulation of immune responses on different levels: on the one hand, by the stimulation of the lymphoid tissue located in the intestine system, it stimulates innate immunity; on the other hand, the interaction between bacterial fragments and receptors (i.e., TLR9 and inflammasomes) placed on the surface of epithelial and immune cells activates specific systemic and local immune responses [25]. When the body’s immune system is no longer able to distinguish self from non-self antigens, it proceeds to attack and destroy its own tissues, by mounting a specific adaptive immune response against self-antigens: this physiopathological model, well known in modern medicine, is called autoimmunity, also defined as loss of self-tolerance. These altered molecular mechanisms involve both the innate and acquired immune system, and lead to altered intercellular communication, underlying the pathogenesis of autoimmune diseases (AIDx) [8,26].

At the base of the mechanisms of recognition of pathogenic signals is the activity of the T and B lymphocytes, which mediate the entire process through the exposure of signals on their surface (Pathogen Associated Molecular Pattern) [8]. When necrosis or apoptosis takes place, endogenous molecules, known as DAMPs (Damage Associated Molecular Patterns), are released and are detected by pattern recognition receptors (PRRs): all this results in a pathological inflammatory response [8,26]_._ This process is also carried out at the intestinal level, where intestinal epithelial cells, through a specific Toll-like receptors complex, Nod-like receptors and helicases, are able to activate the immune response. When abnormal activation of the immune system occurs in response to normally non-harmful stimuli, the autoimmune process takes place [8,26,27,28]. In recent literature and current clinical practice, there is a solid evidence base of strong interconnection between autoimmune processes and psychiatric disorders [29]. Underlying this phenomenon could be an altered antigenic expression of brain proteins, mechanisms of molecular mimicry and production of autoantibodies, that could lead to cross-reaction mechanisms in the brain, as already observed for neurological diseases such as Parkinson’s disease or multiple sclerosis [30,31]. Various mechanisms could lead to the realization of the process of autoimmunity. While genetic susceptibility can have a function in preparing individuals for such abnormal events, it is now clear that environmental factors such as stress, xenobiotic exposure, microbiota dysregulation, and pathogenic infection play a role in modifying, increasing or decreasing, such response [12,31].

When the immune function of the body is impaired, the metabolism of certain metabolites and precursors of neurotransmitters, also produced by the intestinal microbiota, is modified: this is the case of a metabolite of tryptophane (precursor of serotonin) also produced by the intestinal flora, called kynurenine. The metabolism of kynurenine, resulting in the production of kynurenic and quinolinic acid, under conditions of autoimmune alteration, is accelerated, consequently modifying the efficiency of the secretion of GABA and dopamine [32,33], plasticity and synaptic functionality. These two metabolites play a different role in relation to their concentration: for example, adequate levels of kynurenic acid have a neuroprotective role against *N*-methyl-d-aspartate receptor antagonists, whereas increased levels of quinolinic acid could lead to cognitive deficits, as a result of the synaptic dysfunction [34]. According to some authors, the mechanism of excitatory toxicity of quinolinic acid may also affect synaptic plasticity [24,35].

### 1.3. The Impact of Diet on Microbiota and Mental Health

The diet is able to regulate the commensal intestinal flora, influencing the composition of the bacterial diversity in its heterogeneity and proportions. People with higher levels of obesity have more bacteria belonging to the phylum *Bacteroides*, than the normalweight population [36]. In this regard, some studies have shown that low-calorie diets could affect the microbiota to such an extent that the number of *Bacteroides* bacteria could be reduced [36]. Diets rich in unsaturated fatty acids could cause greater reactivity of the immune system and could act as both a systemic and cerebral proinflammatory stimulus, thus favoring greater susceptibility to neurological and psychiatric diseases [37]. Diets with higher fiber content, such as vegetarian ones, promote more intestinal absorption and facilitate digestion by increasing peristaltic activity. Some dietary fibers can be further classified as prebiotic, because they modulate the composition of the intestinal microbiota, bringing an increase in the bacterial population of probiotic microorganisms such as *Bifidobacteria* and *Lactobacilli* [38]. Diets rich in vegetable proteins, such as vegetarian and Mediterranean ones, lead to an increase in the species *Bifidobacterium* and *Lactobacillus*, while those *Bacteroides* and *Clostridium perfringens* decrease [39]. These diets are associated with better intestinal barrier function and reduced risk of inflammation. Higher carbohydrate diets favor an increase in *Enterobacteriaceae*, species that appear to be associated with both intestinal and cerebral inflammation [39,40]. A higher amount of carbohydrates could also seem to determine a reduction in lactobacilli [39,41].

The type of diet seems to be able to influence mental health, regulating the intestinal microbiota and autoimmune processes. The Mediterranean diet, compared to the western one, could favor not only cardio-vascular and metabolic health but also mental health [3,42]. In addition, the type of diet based on the timing of food intake, such as intermittent fasting, could produce different outcomes on mental health [43].

## 2. The Gut Microbiota in Psychiatric Diseases

### 2.1. Schizophrenia

The role of the intestinal microbiota has also been studied in relation to the immunopathogenesis of schizophrenia (SZ). Among the key conditions that the microbiota performs, there is the regulation and increased permeability of the intestinal membrane that could favor the translocation of bacteria in the systemic circulation. Such an event could induce the activation of Toll-like receptors (TLR), resulting in the activation of innate immunity processes. An increased immune activation against dietary proteins, such as wheat gluten and milk casein, and pathogens, such as *T. Gondii* and viruses, has been found in people suffering from SZ [44]. Continuous exposure to antigens may predispose the digestive system to chronic inflammation, endangering the integrity of the intestinal–blood barrier and thereby altering its permeability, with an increased risk of passage into the systemic circulation of bacterial and food peptides and toxic products of bacterial origin. In turn, these, passing into the bloodstream, could give rise to the systemic inflammatory state. Peptides of food origin can have a double impact on the permeability of the intestinal barrier, with a direct effect by modulating tight-junction proteins, and with an indirect effect by cytokine production. In this direction, several studies have confirmed a central role of the immune system in the SZ etiopathogenesis, showing an increased inflammatory state with production of pro-inflammatory cytokines such as interleukine-6 (IL-6) and Tumor Necrosis Factor-α (TNF-α), of E2 prostaglandin inflammation mediator (PGE2), and with an increased COX activity, resulting in the production of cyclooxigenasae-2, detected in plasma of patients during the first episode of psychosis (FEP) [44]. It has been noticed that the structural similarity between the blood–intestinal barrier (BIB) and the blood–brain barrier (BBB) could suggest that the processes that disrupt gastrointestinal localization could similarly disrupt CNS localization [8]. The permeability of endothelial barriers can be influenced both by environmental factors and by genetic factors, such as mutations that compromise the cellular architecture of the barrier [45]. Specific genes have been found to be involved in the constitution of the intestinal barrier, thus being identified as susceptibility elements in the SZ etiopathogenesis. These genes include the protein of the claudin-5 junction, actin, haptoglobin and nitric oxide synthetase [46]. Further genome-wide studies have isolated almost 108 loci associated with psychotic syndromes, as well as loci of human leukocyte antigen (HLA), confirming the immunopathogenic hypothesis in the SZ etiopathogenesis [47,48].

Alteration on microbiota could be the result of several risk factors in psychotic patients: among these, infections and consequent activation of Th17 cells can be found. When a pathogen contacts the host’s immune system, Th17 are stimulated. When activated in response to external stimuli, the same cells have been found to be responsible for gastrointestinal inflammation causing intestinal dysbiosis (an alteration of the normal commensal population). Among infectious species, *Toxoplasma Gondii* is one of the most studied in relation to autoimmune processes that increase susceptibility to SZ, and now considered one environmental risk factor for the disease since, according to pre-clinical studies with rodents, this protozoan could be capable of modifying both behavior and affectivity, increasing anxiety levels [49,50]. These mechanisms could be mediated precisely by the alteration of the microbiota through the pathogenic growth of the commensal flora [51].

Other environmental risk factors for schizophrenia development, and involved in microbiota alteration, include conditions occurring during the prenatal period, like maternal infections during pregnancy. These events could affect neurological development, resulting in an increased risk of developing brain disorders, or induce a pro-inflammatory activation state responsible for metabolic consequences in the long term, such as reduced glycemic regulation and consequent insulin resistance as well as increased body weight (metabolic syndrome-like symptoms) [52]. All these perinatal conditions could induce an inflammatory state responsible for a different neurological development in the offspring, increasing the risk of developing schizophrenia-like endophenotypes, as described in the establishment of the neuropsychoimmunological model known as the “maternal immune activation model” (MIA). Animal studies have been conducted with the administration of agent poly(I:C), a synthetic analogue of a double-stranded RNA, able to induce an inflammatory state in the animal model. This sequence is recognized, by Toll-like receptor type 3, as a pathogen-associated molecular pattern (PAMP) [53]. The purpose of this treatment is to induce SZ-like endophenotypes in the offspring in order to test the neuroimmune development hypothesis of SZ [44,53,54].

Several autoimmune diseases correlate with schizophrenia, some of which have an intestinal involvement and are indirectly linked to alterations in the microbiota. Rheumatoid arthritis was one of the first diseases studied in connection with schizophrenia, revealing an inverse correlation between these two conditions and highlighting the possible autoimmune character of the pathogenesis of SZ [44,55]. Psychosis has been found in association with several autoimmune diseases, such as psoriasis, autoimmune thyropathy, autoimmune hepatitis and, on a systemic level, multiple sclerosis and systemic lupus erythematosus [55].

The first autoimmune disease studied in association with schizophrenia was celiac disease (CD), back in 1953 [56], and then in 1961 [57], when F. Curtis Dohan linked wheat consumption with the onset of the disease: he found out that this association could have been the result of the interaction between the environment and genes [58]. Later, genetic studies on psychotic patients demonstrated a susceptibility role for specific chromosomal regions, such as the 6p21 locus [59]. This one in particular hosts genes for the major histocompatibility complex (MHC) and the human leukocyte antigen (HLA), including a heterogeneous group of genes responsible for immunological mechanisms and mediators of synapse development [60]. In particular, it has been demonstrated that patients affected by CD mostly have heterodimer HLA-DQ2 or haplotype DQ8 [55,61].

Celiac disease is more associated with anxiety disorders than psychotic disorders: in particular, celiac disease seems to correlate more with an increased level of state anxiety than trait anxiety [41,62] and a greater presence of phobic disorders, such as social phobia [63]. The prevalence of state anxiety, compared to trait anxiety, has also been found in IBDs (Inflammatory Bowel Diseases) [40]. As for IBDs, clinical practice shows a high rate of comorbidity with depression and anxiety disorders: this could be explained by the chronic inflammation in association with the immune system’s activation that occurs in IBDs, known as risk factors for mood disorders [64]. Conversely, patients affected by SZ very often have intestinal problems, with strong evidence for the association with irritable bowel syndrome (IBS): all of these could be influenced by gut microbiota, through bacterial translocation [44,55]. According to Gasbarrini et al., gut microbiota could indirectly affect, in predisposed subjects, a neuroinflammatory state responsible for the onset of neurodegenerative diseases, such as Alzheimer’s disease: bacteria could promote inflammation, leading to mechanisms of molecular mimicry and accumulation of β-amyloid peptide (Aβ) in the brain [65].

### 2.2. Bipolar Disorder

The composition of the gut microbiota in patients affected by bipolar disorder (BD) could appear to be different from healthy subjects. BD patients could have a lower proportion of the microorganism *Faecalibacterium* and a larger reduction in the presence of this microorganism could correspond to a worsening of the pathology, with greater alterations in sleep and the onset of psychotic symptoms [66]. DB patients could show a greater representation of the phylum *Actinobacteria*, specifically *Coriobacteria* [67], with higher concentration of Gram-negative bacteria *Prevotella* and *Enterobacter* species, and Gram-positive bacteria *Atopobium* Cluster, *Clostridium*, and *Flavinofractor* [68,69]. Gut microbiota’s composition differs not only between subjects suffering from DB and healthy subjects but also between subjects affected by DB type 1 and DB type 2. The genus *Prevotella* could be more represented in bipolar type 1 patients, while the genus *Collinsella* is more abundant in bipolar type 2 patients [68].

Growing evidence indicates that studying intestinal flora could help us to understand the altered production of neurotransmitters that occurs in these patients, due to the production of neuroactive substances. As mentioned above, this is the case for kynurenine, a neuroactive substance, capable of inhibiting the synthesis of 5-HT [32]; accumulation of metabolites of this substance, such as hydroxykynurenine, has a neurotoxic activity that interferes with the function of neurotransmitters [32,33,34,35]. The immune system could also affect the kynurenine pathway, inducing an abnormal metabolism with consequences on neurotransmitters’ secretion, such as dopamine and GABA [33]. In this regard, it is essential to remember how the onset of DB is strictly related to alterations of the tGABAergic system [24].

GABA can be produced by certain commensal elements of the microbiota, such as *Lactobacillus* and *Bifidobacterium*. Other neurotransmitters involved in the pathogenesis of BD include norepinephrine, which can be synthetized by *Bacillus*, *Escherichia Coli* and *Saccharomyces*, serotonin produced by *Candida*, *Streptococcus*, *Enterococcus* and *Escherichia*, and dopamine by *Bacillus* and *Serratia*, acetylcholine by *Lactobacillus* [69].

The gut microbiota modulates innate immunity as well, through the Toll-like receptors (TLRs) activity: these receptors, widely expressed in the nervous system on the surface of immune cells, neurons and glial cells, are responsible for the detection of antigenic determinants of Gram-positive bacteria and Gram-negative lipopolysaccharides (LPS). When the interaction between these components is realized, several pro-inflammatory cytokines (IL-6, IL-1α, IL-1β and TNF-α) are realised, inducing an inflammatory state in the brain [70].

Further mechanisms that might involve the microbiota in the pathogenesis of the DB are those related to synaptic pruning: this process could be modified by gut microbiota, due to this direct effect on microglial cells [43,44]. As demonstrated in neuroimaging studies, abnormalities in neuronal connectivity due to a deficient synaptic pruning process, especially in the ventral prefrontal and limbic cortex, could be a result of the composition’s changes of gut microbiota in BD patients [66,67,68,69,70,71,72].

As described for psychotic disorders, IBS along with several intestinal diseases are commonly reported in patients affected by mood disorders: what further complicates the clinical management of these conditions is the high rate of psychiatric problems, with a strong prevalence of anxiety and depression [69]. There is evidence of an indirect correlation between mood disorders and intestinal autoimmune diseases, such as celiac disease, and it has been noticed that an improvement in mental health through psychological support would lead to a better outcome of celiac disease [73]. Studies have shown that BD patients often experience intestinal inflammation, during mood episodes, with higher levels of pro-inflammatory cytokines [73,74,75,76]. Changes in the pathways of tryptophan metabolism, mostly regarding kynurenic pathways, appear to play a role in the BD pathogenesis [77]. Tryptophan is transformed into excitatory neuroactive compounds, able to antagonize n-methyl-d-aspartate receptors (NMDARs), such as kynurenic acid [78,79]: excessive concentration of these metabolites has been observed in BD patients [80].

Other comorbidities straddling between mood and gastrointestinal disorders are those induced by alcohol use, which has markedly increased during the COVID-19 era; social isolation and external stressors have strengthened this correlation [81]. Alcohol could affect microbiota, while a healthy microbiota could contribute to a regression of liver pathologies, such as steatosis [82].

An additional element of correlation between microbiota and BD is the effect that antibiotics can have on some of these patients. Although rare, antibiotics can induce a maniacal episode, sufficient to lead some authors to speak of “antibiomania” [83].

In BD patients, interventions through a balanced and healthy diet have been shown to increase the treatment response rate. The use of antioxidants, vegetable fibers and B-group vitamins could decrease the risk of weaving depressive symptoms [84], while short-chain fatty acids could have positive effects on the cognitive sphere, improving neurogenesis and synaptic plasticity [68,85].

### 2.3. Unipolar Affective Disorder

Of all psychiatric disorders, mood disorders have always been of great interest in research because of their impact on the general population. According to the latest estimates of the Global Health Data Exchange, depression affects about 280 million people worldwide, about 5 % of the adult population (2021) [86]. A significant increase in the diagnosis of depression in recent years was due to the COVID-19 pandemic and its consequences on social, economic and health issues. Great attention has always been paid to depressive disorders for their impact on the quality of life of patients and family members, and for the high risk of suicide. For decades, we have been focusing on the physiopathogenetic mechanisms that could be behind the major depressive disorder (MDD), with considerable difficulty given the inter-individual variability and the heterogeneous result of the gene–environment interactions, well described in the medical literature. In this regard, a renewed and recent interest has been directed to the brain–gut–immune system axis: existing data reveal that intestinal bacteria can modulate immune response to stress and external stimuli through changes in the development and function of the hypotalamic–pituitary–andrenal axis (HPA) [6,87,88,89]. The resulting dysregulation of the HPA axis leads to an increased concentration of cortisol and pro-inflammatory molecules, correlated with depressive and anxiety disorders [88,90]. Conversely, this proinflammatory state can aggravate microbiota itself causing harmful consequences on gastrointestinal homeostasis, leading to systemic inflammation: higher circulating cortisol levels and pro-inflammatory molecules increase the intestinal barrier permeability, facilitating the access to the bloodstream for Gram-negative bacteria inducing chronic inflammation in the central nervous system, with consequences on emotional processing and mood regulation [87,91]. Again, this evidence shows the strong connection between microbiota-driven inflammation and psychiatric disorders (as already seen for IBS), in particular anxiety and depression [92].

The recent literature shows that the gut microbiota may differ significantly between subjects suffering from MDD and healthy subjects [4,93]. As in other psychiatric disorders, an attempt has been made to define a possible microbial profile in the depressed patient, where a decrease in the concentration of alpha and beta diversity is observed: while on one hand there is a reduction in the concentration of *Firmicutes*, *Bacteroides* and *Proteobacteria*, on the other there are increased levels of *Actinobacteria* and *Fusobacteria* [4,93], *Prevotellaceae* and *Lachnospiraceae* [94,95,96]. Decreased concentrations of *Bifidobacterium*, *Firmicutes*, *Lactobacillus*, *Faecalibacterium* and *Ruminococcus* and increased concentrations of *Proteobacteria*, *Bacteroides* and *Prevotella* have been found in the gut microbiota of depressed patients [97,98].

Valles-Colomer et al. describe increased numbers of Flavonifractor and a depletion of *Coprococcus* and *Dialister* [99]. Other studies have observed a correlation between *Faecalibaterium*, *Alistipes* and *Ruminococcus* with MDD [100]. An element common to all recent reviews confirms the overabundance of microbial species, such as *Actinobacteria* and *Enterobacteriacaee*, able to increase a proinflammatory profile at the intestinal level, with systemic repercussions, and a reduction in protective species such as *Faecalibacterium* and *Firmicutes* [94,96,101,102]. To complicate this picture, the microbial intestinal profile of an individual is also affected by environmental factors such as diet [103] and geographical area [104], in addition to genetic factors [105] and age [106]. Several communication mechanisms could be considered as connected to the gut–brain axis, such as the immune system, the vagus nerve and the modulation of neuroactive molecules produced by the microbiota itself [107]. It has been observed that the bacteria that make up the gut microbiota can alter the neurotransmitting composition of the individual through their production or consumption; consequently, it has been outlined that interventions aimed at modifying the intestinal microbiota are able to alter the levels of some neurotransmitters. Among these, dopamine is one of the best known and studied in the psychiatric field, involved in reward mechanisms, and a precursor of catecholamines, such as epinephrine and norepinephrine; the latter in particular plays a fundamental role in the mechanisms of arousal and alert, and as more recently discovered, in cognition, memory and attention [108,109,110]. Alteration of these neurotransmitters’ concentration has been detected in individuals suffering from bipolar depression and unipolar depression, with higher norepinephirine levels in the blood plasma and urine [111]. Other studies emphasize the key role of dopamine as well, showing that dopamine antagonists aggravated depressive symptoms, while dopamine agonists have an antidepressant-like effect [112,113,114]. The same effect is exploited by drugs with dopamine reuptake activity, such as bupropion and venfloxacin [112,113]; additionally, an increased level of homovanillic acid, the main metabolite of dopamine, has been found in the mesolimbic and mesostriatal area in individuals affected by MDD treated with transcranical magnetic stimulation (TMS) [115]. Moreover, the mesocorticolimbic pathway could be influenced by gut microbiota, since the dopaminergic system strictly works with the microbiota itself [116]. What makes it possible is the contribution of the HPA axis [117], the immune system [118] and the vagus nerve [22,119]: the latter, when stimulated, increases the levels of dopamine in the brain [120]. It has also been observed how bacteria regulate and are regulated by the levels of these catecholamines: when dopamine and norepinephrine levels are increased, some pathogenic bacteria, such as *Escherichia Coli* O157:H8 (EHEC), proliferate [121] and increase their virulence factors, such as motility and biofilm formation [122]. Increased grown rates with such catecholamines are also observed in vitro for other pathogenic bacteria such as *K. Pneumoniae*, *P. Aeruginosa*, and *S. Aureus*. [123].

In addition, by regulating catecholamine synthesis, gut microbiota determines the growth of other bacteria [107], such as *Serratia*, *Morganella*, *Klebsiella*, *Escherichia C and Lactobacillus* [124,125], which in turn produce dopamine. More recently, it has been observed that a gastrointestinal bacterial depletion induced by antibiotics in murine models led to increased levels of levodopa and its precursor homovanillic acid (HVA) in the prefrontal area, and a reduction in HVA in the hippocampus, as well as a reduction in the HVA/dopamine ratio in the amygdala and striatum [126]. Other studies have highlighted the beneficial role of intestinal commensal bacterial flora compared to the dopaminergic system [127,128,129]: administration of *L. Paracasei* PS23 (both live and heat-killed) decreased levels of 3,4-dihydroxyphenylacetic acid (DOPAC) and HVA, without reducing levels of (DOPAC+ HVA)/DA in the hippocampus of the mice model exposed to early life stress.

Like dopamine and catecholamines, serotonin (5-HT) is also largely involved in gut microbiota-mediated mechanisms: more than 90% of the total 5-HT of an individual is produced in the gut [130,131], as its production was observed in several bacterial species, such as *Candida*, *Escherichia*, *Streptococcus*, *Enterococcus*, *Klebsiella P.*, *Lactobacillus Plantarum*, and *Morganella M* [125,132,133,134]. The microbial production of serotonin at the intestinal level would be regulated by the secretion of butyrate, able to stimulate the production and release of 5-HT from Enterochromaffin cells (ECs) [130,130].

### 2.4. Anxiety Disorders

As is well known, anxiety disorders are a nosographic category present transversely in current clinical practice; they frequently occur in comorbidity with disorders of mood, shape and content of thought or eating behavior disorders, or constitute the principal diagnosis. Years of research and revisiting of scientific literature have attributed a central role to the development of such manifestations to the HPA axis [92]. Hormonal changes affecting this axis have been associated with psychiatric disorders and particularly anxiety disorders and stress-related disorders [4]. When a stressful event occurs, there is an activation of the HPA axis, which begins with the release of corticotropin-releasing hormone (CRH) by the hypothalamic paraventricular nucleus (PVN). This, in turn, induces the release of the adrenocorticotropic hormone (ACTH) into the bloodstream; from here, the ACTH reaches the adrenal gland, where it induces the release of cortisol, which, along with epinephrine and norepinephrine, induces the response “fight or flight”. This response translates into a metabolic level with an increase in gluconeogenesis, suppression of the immune response, and increased lipid and protein metabolism [4,92,93]. This mechanism is, in turn, subjected to a negative feedback mechanism, since increased levels of cortisol in the bloodstream are captured by receptors located at the hypothalamic and hippocampal level, which induce a downregulation of the stress response [91]. If such stress-induced responses occur during intrauterine life, they are able to affect the development and the activity of the HPA axis, including chorionic production and its release in response to external stimuli. Overstimulation of this axis can impact the individual’s behavioral development and emotional regulation, leading to different forms of psychopathology [6,129]. The proper development of this communication pathway could be linked to the intestinal microbiota: recent studies have shown how stressful events during intrauterine life and early childhood are associated with intestinal dysbiosis in the unborn child and in the mother [135,136,137,138]. This increased susceptibility in the unborn child is also determined by the immaturity of both the HPA axis and the microbiota itself: thus, stressful events can be so impactful as to affect the intestinal microbiota, leading to dysbiosis [135,136]. In this complex process, several studies have highlighted the role that cortisol could play in the intestinal microbiota: in one study, it was observed that maternal stress and increased salivary cortisol levels during the last months of pregnancy correlate with a disruption of the intestinal microbiota of the unborn child, with persistence of such alterations over a period of at least 16 weeks [139]; in another study, it was found that cortisol could directly determine changes in the commensal microbial profile, for a shift in gene expression [140]. Jašarević et al. suggested that alterations in the microbiota were associated with increased concentrations of circulating corticosterone in response to stressful events, and that the transmission of stressed-altered maternal microbiota had long-term effects on gene expression at the level of the PVN [141]. Neufeld et al. found that the microbiota could regulate the serotonin system in the brain, influencing the HPA axis and modifying gene expression at hippocampal and hypothalamic levels [142].

It is therefore evident that events that occur during the intrauterine life can determine changes at the level of the microbiota, with consequences on the development of correct adaptive responses to stress in subsequent years of life.

### 2.5. Eating Disorders—Anorexia Nervosa and Bulimia Nervosa

Anorexia nervosa is the most known eating disorder and is characterized by a gradual and increasingly important reduction in calorie intake, often accompanied by excessive and compulsive exercise, leading to progressive body weight loss (DSM-5) [143]. Furthermore, there is alteration in the way in which the corporeality is experienced and scarce insight. Hilde Bruch, in 1962, described the distortion of the body image, associated with malnutrition, as pathognomonic of the disease [144]. This condition is still more common among women, with an estimated prevalence rate of 0.3–1% among women and around 0.1–0.3% among men in western countries, despite an alarming increase in prevalence rate in males in the last few years. This represents a public health issue, considering that anorexia and bulimia nervosa have the highest mortality and suicides rates among all psychiatric disorders [145,146,147]. Among all psychiatric disorders, eating disorders are certainly those where the role of the brain–intestine axis is most evident, due to the important and debilitating physical conditions in which patients find themselves (chronic calorie restriction, imbalance and deficit of macronutrients, osmotic disturbances, etc.) [148]. These conditions lead to important alterations of the intestinal microbiota: in many studies, dysbiosis states are found with an increased concentration of bacteria of the species *Clostridia*, *Enterobacteriaceae* and *M. Smithii* and reduction in the species *Roseburia* [149,150,151]. In other studies, a reduction in alpha diversity has also been observed in conjunction with body weight reductions and caloric restriction, along with decreased levels of Firmicutes and short-chain fatty acids (SCFAs). Moreover, weight recovery has been associated with increased SCFAs levels and a re-establishment of the *Firmicutes/Bacteroides* ratio. In addition, one study showed increased levels of caseinolytic proteinase B (ClpB), produced by *E. Coli*, which in turn stimulate autoimmunity response: it has recently been demonstrated that Cl, pB and other dietary antigens are able to stimulate autoantibody (autoAb) formation, leading to cross-reaction with neurotransmitters and hormones that regulate the centers of appetite [152,153,154]. The activity of these centers is complexly regulated by an elaborate neuroimmunoendocrine communication system, with the microbiota regulating the activity of adipose tissue and general homeostasis [155,156,157]. A subversion of this communicative system is present in anorexia and bulimia nervosa, due to an alteration of the cholinergic, dopaminergic, adrenergic and serotoninergic system [154]. For example, the structural similarity between the bacterial protein ClpB and the anorexigenic/anxiogenic hormone alpha-MSH would lead, through molecular mimicry mechanisms, to the formation of antibodies directed against human alpha-MSH [157]. Increased levels of IgM autoantibodies against alpha-MSH have been found in patients affected by anorexia nervosa. Another study illustrated a reduction in IgG autoantibodies’ concentration directed against alpha-MSH in obese patients, and increased formation in anorexic and bulimic patients: in the latter, the formation of immunocomplexes between IgG and alpha-MSH could induce a state of chronic activation of the melanocortin system intercalated in the pathways of appetite regulation [152,154,155,156], through stimulation of the MC receptor type 4 (MC4R) [157]. Other recent studies have revealed other classes of altered autoantibodies in patients suffering from eating disorders, especially in patients affected by anorexia nervosa, including increased levels of IgG against ACTH [158,159,160]; IgG leptin-neutralizing autoantibodies have been identified in healthy individuals with reduced BMI, while diminished affinity of the same autoantibodies has been found in obese patients [154]. Alongside these modifications, an altered expression of pro-inflammatory and anorectic cytokines such as IL-6, IL-1beta and TNF-alpha has been associated: among these, TNF-alpha and IL-1beta influence the expression of some neuropeptides, in turn associated with anxiety disorders and anorexia nervosa [161,162,163]. To confirm this hypothesis, there are studies that highlight the positive effects of monoclonal anti-TNF-alpha therapy in cases of anorexic patients in comorbidity with Crohn disorder and juvenile idiopathic arthritis [164,165].

As mentioned above, the intestinal microbiota transforms the proceeds of the diet into a large variety of products including vitamins, amino acid derivatives and SCFAs, able to modulate the permeability of the blood–brain barrier (BBB) [166,167,168]: SCFAs, in particular, increase the expression of tight junctions’ complexes such as occluding, zonulin and claudin-5, thereby reducing its permeability [169]. SCFA can also mediate appetite reduction through the increase in glutamatergic transmission of the POMC/CART and GABAergic pathway, by reducing NPY/AGRP to the hypothalamic arcuate nucleus [170]. In addition, weight loss and fasting could induce a reduction in gastroenteric permeability, while increasing BBB permeability through the release of SCFA and ketone bodies. The same changes have also been described in immuno-related Paediatric Autoimmune Neuropsychiatric Disorders Associated with Streptococcal Infections, known as PANDAS [171].

## 3. Diet and Its Implications

### 3.1. The Role of Diet in Microbiota Regulation

Diet can influence the microbiome, through changes in the epithelial permeability of the intestine, with resulting alteration of the absorption of nutrients: this enhances the risk of circulating bacterial toxins and peptides, with subsequent chronic inflammatory state and activation of neural pathways, altering the activity of the CNS [24]. Diet has a central role in the composition of the microbiota, and variations in the dietary regime involve, indeed, changes in the intestinal commensal population [172]. Short-term dietary changes result in reversible and short-term changes in the human microbiota [36], while prolonged dietary changes result in a more significant and stable change in the commensal flora. An example of this dietary style–composition ratio of the microbiota is valid for states of prolonged malnutrition, which have a significant and constant impact on the gut microbiotic population, while short-term variations in the diet modify the microbiota with the possibility to return after a short time to its starting composition. Another example is the infant gut microbiota, which changes as the infant switches from liquid to solid foods [173]. In the newborn, the dietary style is what influences microbiotic composition the most, along with infections, genetics, lifestyle, delivery methods and the possible use of antibiotics [3]. Changes in gut microbiota depend on a healthy or unhealthy diet (Table 1). Other elements that could contribute to this inflammation process are feeding time, with regard to intermitting fasting or limited feeding time, and circadian rhythms [43]. The composition of the microbiota varies according to the weight of the person, too: the commensal flora of obese people presents a lower number of bacteria belonging to the phylum *Bacterioides* than the normal-weight ones, so that according to some studies this proportion could be restored through a low-calorie diet [36].

### 3.2. The Role of Single Nutrients

Excessive accumulation of Saturated Fatty Acids (SFA) could act as a proinflammatory signal both in the peripheric and central nervous system, whereas other studies show that some psychiatric patients, such as bipolar disorder patients, would have significantly reduced levels of docosahexaenoic acid (DHA) [178]. Palmitic, lauric and stearic acid SFA have been shown to activate TLR4 receptors of intestinal macrophages [179], promoting nuclear factor kB disinhibition, with subsequent production of proinflammatory cytokines, like interferon-γ (IFN-γ), TNF-α, IL-1β and IL-6. In addition, fatty acids contribute to bacterial flora alteration by increasing Gram-negative microbes’ concentration with production of Lipopolysaccharides (LPS), a natural ligand for TLR4 [180], and also by increasing intestinal permeability with easier access for LPS and bacteria to the bloodstream, causing inflammation. Inflammation could spread to the brain, where neuroinflammation could precede systemic inflammation. Neuroinflammation could first affect neural networks in the hypothalamus and in the nucleus tractus solitarius, with subsequent dysregulation in energy balance and excessive food consumption [181], extending to amygdala and hippocampus, altering memory and learning processes with an increased risk for the development of neurodegenerative diseases [1]. Studies on a murine population have shown antidepressant and anti-inflammatory effects and improved social deficits in the presence of butyrate, a Short-chain fatty acid (SCFA) produced by dietary fibers fermentation. Effects of its activity can be found on a genetic level, since it has been shown to contribute in the modulation of histone deacetylase inhibitor within the monoaminergic pathway. Again, among psychiatric disorders, lower levels of butyrate have been detected in patients affected by anorexia nervosa [182].

#### 3.2.1. Fibers

Bacteria use fibers to produce Short-chain fatty acid (SCFAs), which enter the bloodstream and easily spread through the blood–brain barrier. Their anti-inflammatory effect is finally accomplished once they have reached the brain, where they interact with microglia [182,183]. Among the functions related to the human microbiota, some fibers also perform the activity of bacterial fermentation, including pectin, xanthan and guar gum, and b-glucan. Fermentable dietary fibers constitute an important source of energy for intestinal microbes, considering the fact that they can also alter microbiota composition through a cross feeding process, which consists of the production of metabolites from bacteria used for the growth of other commensals [184]. Due to their modulation effect of intestinal flora composition, dietary fibers can be considered as prebiotics or as non-viable food components: galactooligosaccharide and polydextrose, for example, increase the levels of *Lactobacilli* and *Bifidobacteria*, which in turn suppress the growth rate of other pathogenic microbes [185]. In addition, it has been shown by further studies that they seem to improve anxiety symptoms and to improve cognition [186]. Inulin enriched with fructooligosaccharides also improved cognitive performance in murine species [3,187].

#### 3.2.2. Vitamins

Vitamin deficiencies can alter cognitive abilities and induce neurodegeneration by reducing the number of hippocampal neurons [188]. Most vitamins cannot be synthesized but must be obtained by nutrients, while some vitamins can be obtained by additional biochemical pathways, such as the production by the intestinal bacterial flora of vitamin K. The Vitamin K family includes two main members: vitamin K1, called phylloquinone, and vitamin K2, called menaquinone. The first one can be found in vegetable oils and green leafy vegetables, while the second can be found in animal products, such as eggs and meat [188]. Menaquinone could be directly produced by intestinal bacterial flora or indirectly by the metabolism of vitamin K1 [189]. It has also been noticed that increased levels of *Bacteroides* and *Prevotella* in the intestinal flora are associated with an increased concentration of menaquinonic forms of vitamin K [189]. Menaquinone is the most present form in the healthy CNS, and also vitamin K administration, in menaquinonic form, seems to prevent the onset of anxiety and depression in rats [190].

Retinol, mostly known as Vitamin A, induces cellular differentiation and neural plasticity in the CNS, with involvement in immunological processes and vision. Chronic intoxications of vitamin A are associated with symptoms of anxiety, depression and cognition. Administration of vitamin A supplements can alter intestinal flora, with an increasing presence of the family *Lactobacillaceae*, while vitamin A depletion induces an inflammatory state in the colon [191].

Vitamin D plays a fundamental role in fetal neurodevelopment so much so that the introduction of vitamin D before weaning can prevent learning and memory deficits and locomotion disorders. Vitamin D deficiency of its D receptor is associated with a reduction in the number of butyrate-producing flora, including *Firmicutes*, and with a higher number of *Proteobacteria*, such as *Bacteroidetes* [3]

#### 3.2.3. Carbohydrates

Among the carbohydrates that have the greatest impact on the microbiota are refined carbohydrates, such as white bread, white rice, pasta, and starch [179]. Studies on murine species have shown that higher consumption of refined carbohydrates could increase *Enterobacteriaceae* levels, with connected intestinal and cerebral inflammation, and decrease *Lactobacillus* associated with SCFA transport [192]. In addition, the correlation between a high blood sugar value and susceptibility to neuroinflammation is already known, resulting in deficits in cognitive performance in the long term [1].

#### 3.2.4. Minerals

Iron deficiency has been related to lower levels of butyrate production by intestinal bacteria, while iron excess correlates with higher levels of *Bacteroidaceae* and *Lachnospiraceae*, which in turn lead to increased production of propionate. Zinc, the most widespread metal ion in the nervous system, is known to be involved in neurogenic and synapthogenic processes: zinc excess, as well as zinc depletion, causes alteration in microbiota diversity and composition [193].

#### 3.2.5. Polyphenols

Polyphenols are natural organic substances. The most common polyphenols in nature are flavonoids, tannins, lignins, anthraquinones and melanins. These are produced by plants, animals, fungi and bacteria. We can split them into two groups: flavonoids and non-flavonoids [194]. Among phenolic compounds that mostly affect the gut microbiota, there are resveratrol contained in red grapes, quercetin contained in oranges, tangerines, apples and vegetables, and hydroxyglycol found in extra virgin olive oil [195,196,197]. These compounds act through antioxidant and anti-inflammatory properties [197], and affect the activity of the intestinal microbiota by stimulating the growth of microorganisms such as *Lactobacilli* and decelerating the growth of others such as *Enterococci* [198]. Phenols in olive oil, such as hydroxystyrene, increase *Bifidobacteria* levels [199]. According to studies on murine models, resveratrol on the one hand inhibits the intestinal growth of bacteria belonging to the species *Enterococcus* and *Firmicutes*; on the other, it promotes greater colonization by *Lactobacilli* [200]. Quercetin, also according to murine studies, would favor the increase in *Lactobacilli* and *Clostridia*, while it would inhibit *Enterococci* [201]. Other polyphenols that contribute to the modulation of the intestinal microbiota include hydroxyinnamic acid (CGA). Contained in coffee, CGA is metabolized by the intestinal flora into aromatic acid after hydrolysis to quinic acid [202]. As well as caffeine itself, CGA has antioxidant and anti-inflammatory effects [203,204].

### 3.3. Different Types of Diets

#### 3.3.1. Western Diet

The Western diet is characterized by a high intake of sugars, saturated fats and refined cereals, and is linked to decreased levels of intestinal microbiotic diversity: reduced number of members of the phylum *Bacteroidetes* has been found, along with higher levels of *Proteobacteria* and *Firmicutes*. The altered bacterial flora, as mentioned above, affects brain functioning and structure [3]. Moreover, an increase in fatty acids levels leads to pro-inflammatory state, through the already-mentioned effect of altered microbiota on intestinal wall permeability, with the circulation of bacterial and infectious peptides, as well as the production of LPS that activates the immune response by binding to the TLR4 receptor of intestinal macrophages. As previously mentioned, this would extend to the brain, resulting in neuroinflammation and neurodegeneration processes, thus inducing increased susceptibility to psychiatric and neurological diseases [1,174]. Prolonged exposition to the Western diet has been associated with cognitive impairment and the development of depressive symptoms [205].

#### 3.3.2. The Mediterranean Diet

Among all types of diet, the Mediterranean diet is known worldwide for the beneficial effects on health on a cardiovascular and neurocognitive level [3]. It is characterized by a good intake of vegetables, olive oil, fruits and a moderate amount of bread, pasta, dairy products and meat. Diets rich in vegetable proteins lead to an increased diversity of microbiome composition, with a decreased concentration of *Firmicutes*, *Proteobacteria* and *Clostridia* (*C. Perfringens* in particular), and an increase in *Bifidobacteria* and *Lactobacillus* [39]. This kind of diet correlates with reduced inflammation levels, decreased cytokines release and a greater modulation on intestinal permeability [39]. Due to the richness in saturated fatty acids composition, such as omega-3 (especially docosahexaenoic acid (DHA), primary structural component of the lipid-doubled layer of neuronal membranes) and omega-6, the Mediterranean diet plays a key role in the nervous system, with modulation of synaptogenesis and neurogenesis [175]. DHA has also been linked to regulation of glutamate, serotonin and dopamine neurotransmission: diets rich in fish oils, providing DHA, have been shown to significantly improve behavioral symptoms in patients with ADHD [101,206]. The antioxidant effects of diets rich in omega-3 have been suggested by numerous studies, by preventing protein, lipid and DNA oxidation, thus suggesting the central role of this diet in the management of cardiovascular and psychiatric diseases [207].

#### 3.3.3. Vegetarian and Vegan Diet

According to several studies, heterogeneity of commensal intestinal microbiota could be associated with obesity levels and BMI, and to the compliance of the arterial wall. The association with the latter could be explained by the key role of intestinal microbiota in systemic inflammation [208]. A greater bacterial variability could reduce systemic inflammation, leading to an improvement in arterial structure [209]. In this context, vegetarian and vegan diets may play a central role, due to the positive effect of a long-term diet based on vegetables and fruits, resulting in higher variability in microbiota composition [210]. Several studies highlighted that obese patients have decreased levels of *Firmicutes* and an increased level of *Proteobacteria* and *Enterobacteraiceae* [211]. Conversely, a decreased representation of *Enterobacteriaceae* has been demonstrated in vegetarian patients. Diets rich in fibers contribute to lower intestinal pH, lowering the growth rate of pathogenic bacteria such as *Enterobacteriaceae*. In conclusion, vegan and vegetarian diets seem to stimulate the growth rate of protective microbial species in the inflammation state, such as *Bacteroides* and *Prevotella* and a reduction in *Bacteroides Fragilis* and *Clostridium* [176,177,212], and seem to induce epigenetic changes with a subsequent decrease of risk factors for chronic inflammation.

### 3.4. The Use of Probiotics

Some probiotics affect the activity of the microbiota and the physiology of the brain. Among these, *Lactobacilli*, *Bifidobacteria*, *Saccharmoyches* and *Enterococcus* are the most used, although it should be remembered that health benefits depend on bacterial strain rather than bacterial species [213]. Probiotics could be found in dairy foods such as yogurt, kefir and cheese, while others derive from beer, sauerkraut, chocolate, miso and olives [214]. The literature highlights the presence of probiotics in non-dairy fermented products such as cereals, corn, millet, legumes and soy. The selection process of bacterial strain follows specific criteria: first of all, it must not be pathogenic or toxic [215], and strong enough to reach and colonize the intestinal trait without being attacked by the immune system [216]. In addition, probiotics should be able to produce antimicrobic products to be used against pathogenic agents. Some of them can positively modulate the immune system, preventing both alterations of the intestinal microbiota induced by stress, as well as behavioral and neuropsychiatric changes. In conditions of alteration of microbiotic homeostasis, studies on murine species have shown that there is a reduction in hippocampal cell growth with increased anxiety symptoms [217]. Further studies, conducted on human species, pointed out that integration of probiotics in the diet has positive effects; these could increase the growth rate of *Bifidobacteria* and induce a lower response to cortisol, a hormone closely related to anxiety and depression [217]. Further studies have shown that the use of probiotics leads to a decrease in the state of attentive vigilance [218]. Supplementation with trans-galatooligosaccaridi (trans-GOS) could favor the growth of *Bifidobacteria* and improve anxiety symptoms in IBS. Among all probiotics, *Bifidobacteria* and *Lactobacillus* have been shown to significantly reduce anxiety and depressive symptoms, along with stress-induced responses [32,219]. Other beneficial effects of their use on our organism are the prevention of lactose intolerance and caries formation, reduced possibility of developing IBS and gastrointestinal inflammatory diseases and protection from bacterial diarrhea. Extra-intestinal outcomes have been shown too, such as reduced risk of developing osteoporosis, urinary tract infections and allergies, and reduced plasmatic levels of cholesterol, along with a protective role against cancer [1].

### 3.5. The Fecal Transplant

The centrality of the gut–brain axis is confirmed by the impact of fecal transplantation on mental health. It consists of the injection of filtered feces from a healthy donor to a patient, and is a medical practice known since the fourth century in China where it has been used for the treatment of food poisoning, up to the present day, where fecal transplantation is more effective in treating *Clostridium Difficile* infections than different antibiotic therapies [220]. Patients suffering from Chron’s disease could have benefited from fecal transplantation not only in terms of physical health but also in terms of a better psychological state of health, especially in the sexual sphere. Further neuropsychiatric pathologies, which have seen an improvement in specific symptoms following fecal transplantation, are those of neurodevelopment, especially the autism spectrum disorders [221].

## 4. Conclusions and Future Directions

An analysis of the recent literature shows that a dysregulation of the gut–brain axis is evident in the various major psychiatric disorders, as confirmed by human and animal studies (Table 2). The importance of the gut microbiota to mental health is now consolidated by vast evidence. On the one hand, external environmental factors and predisposing genetic factors lay the foundations for the interindividual variety of the intestinal microbiota; on the other hand, the microbiota itself regulates the production of certain neurotransmitters that can modulate the activity of the central nervous system. Stressful situations can have a great impact on the composition of the gut microbial community, activating the immune system with production of IL-6 and IFN-γ, and modulating metabolic response by decreasing SCFAs levels. Activation of the immune response resulting in changes in the microbiota may induce changes in the intestinal barrier, with increased levels of pro-inflammatory cytokines entering the bloodstream. From the periphery, toxic metabolites resulting from microbiota alterations and pro-inflammatory cytokines can cross the BBB, leading to a change in the pro-inflammatory state at the brain level. Higher levels of inflammation in the brain lead to structural changes in glial cells influencing neural pathways involved in learning, memory, mood regulation and emotions, which could contribute to the onset of several neuropsychiatric conditions.

The composition and function of the microbiota seem to be strongly subject to numerous external factors such as diet, socio-economic conditions, the use of drugs (especially antibiotics) and the use of probiotics. Given the current scientific evidence linking the microbiota with the activity of the CNS, the regulation of these factors could determine the constitution of a healthy microbiota, with beneficial repercussions on mental health, especially in the circuits that regulate the affective and cognitive sphere.

A growing interest in this field has been shown both in terms of mental health prevention and intervention: therapeutic modulation of gut microbiota using pre- and probiotics may be useful in disorders involving disorders of the microbiota–gut–brain axis [22]. Prebiotics can benefit both the intestinal mucosa and systemic immunity as they reach the hydrolyzed large intestine and stimulate the growth of useful intestinal microbiota [119]. Probiotics could restore intestinal permeability by improving [120] mucosal barrier function.

Although the pharmacological approach to psychiatric pathologies plays a central role, behavioral interventions have proven to be a great support: among these, in recent decades more attention has been paid to dietary interventions. Given the key role of inflammatory processes in many diseases of the CNS, the possible therapeutic use of diet in mental health has been considered with greater interest. This seems to be supported by the potential direct effects on inflammation thanks to antioxidant substances such as polyphenols or anti-inflammatory molecules such as Omega-3 fatty acid, and direct effects on functional modulation through amino acids or B vitamins. Interventions such as these have aroused sufficient interest to frame a new branch, called “nutritional psychiatry”.

Future research programs should be focused on finding individual and personalized strategies for mental health, considering both genetic and environmental factors, such as age, gender and comorbid medical conditions, with greater attention to diet and nutrition.

## Figures and Tables

**Table 1:** The role of diet in microbiota regulation.

Type of Diet	Impact on Microbiota	References
Western diet	Decreased levels of intestinal microbiotic diversity (reduced number of members of the phylum *Bacteroidetes* has been found, along with higher levels of *Proteobacteria* and *Firmicutes*).	Tengeler et al., 2018 [3]
	Increase in fatty acids levels leads to pro-inflammatory state, through the already-mentioned effect of altered microbiota on intestinal wall permeability (increased susceptibility to psychiatric and neurological diseases).	Evrensel et al., 2015 [174]
Mediterranean diet	Increased diversity of microbiome composition, with a decreased concentration of *Firmicutes*, *Proteobacteria* and *Clostridia*, and an increase in *Bifidobacteria* and *Lactobacillus*.	Singh et al., 2017 [39]
	Reduced inflammation levels, decreased cytokines release and a greater modulation on intestinal permeability.	Tanaka et al., [175]
Vegetarian and vegan diet	Decreased representation of Enterobacteriaceae and increase of *Bacteroides* and *Prevotella*	do Rosario et al., 2016 [176]
	Reduction in *Bacteroides Fragilis* and *Clostridium* with subsequent decrease of risk factors for chronic inflammation	Ferrocino et al., 2015 [177]

**Table 2:** The composition of the gut microbiota in patients affected by psychiatric disorders.

Psychiatric Disorder	Impact on Microbiota	References
Schizophrenia	Increased immune activation against dietary proteins and pathogens and continuous exposure to antigens predisposing the digestive system to chronic inflammation; alteration of permeability of the intestinal blood barrier and an increased risk of passage into the systemic circulation of bacterial and food peptides; increased inflammatory state with production of pro-inflammatory cytokines.	Caso et al., 2016 [44]
	Stimulation of Th17 cells in response to external stimuli, with consequent gastrointestinal inflammation causing intestinal dysbiosis. Maternal infections during pregnancy inducing a pro-inflammatory activation state responsible of metabolic consequences in the long period (reduced glycemic regulation, insulin resistance, increased body weight). Association of schizophrenia with irritable bowel syndrome influenced by gut microbiota, through bacterial translocation.	Torrey et al., 2012 [50] Labouesse et al., 2015 [52] Severance et al., 2016 [55]
Bipolar Disorder	Lower proportion of *Faecalibacterium* corresponding to a worsening of the pathology.	Painold et al., 2017 [66]
	Greater representation of the phylum *Actinobacteria*, with higher concentration of *Prevotella* and *Enterobacter* species, and gram-positive bacteria *Atopobium Cluster*, *Clostridium*, *Flavinofractor*. *Prevotella* more represented in bipolar type 1 patients, while *Collinsella* more abundant in bipolar type 2 patients. Production of neuroactive kynurenine, capable of inhibit the synthesis of 5-HT and interfere with the secretion of dopamine and GABA. Synaptic pruning could be modified by a direct effect of gut microbiota on microglial cells, especially in the ventral prefrontal and limbic cortex.	Gondalia et al., 2019 [69] Lucidi et al. 2021 [68] Schwarcz et al., 2017 [33] Strakowski et al., 2012 [72]
Unipolar Affective Disorder	Intestinal bacteria can change function of the hypotalamic–pituitary–andrenal axis (HPA) leading to an increased concentration of cortisol and pro-inflammatory molecules: the proinflammatory state increases the intestinal barrier permeability, facilitating the access to the bloodstream for gram-negative bacteria and inducing chronic inflammation in the central nervous system. The possible microbial profile in depressed patients can be defined by a reduction in the concentration of *Firmicutes*, *Bacteroides* and *Proteobacteria*, or by increased levels of *Actinobacteria* and *Fusobacteria*, *Prevotellaceae* and *Lachnospiraceae*.	Li et al., 2019 [91] Barandouzi et al., 2020 [93]
	Decreased concentrations of *Bifidobacterium*, *Firmicutes*, *Lactobacillus*, *Faecalibacterium* and *Ruminococcus* and increased concentrations of *Proteobacteria*, *Bacteroides* and *Prevotella* in the gut microbiota of depressed patients.	Liu et al., 2016 [97]
Anxiety Disorders	Stressful events during intrauterine life and early childhood are associated with intestinal dysbiosis in the unborn child and in the mother. Alterations in the microbiota associated with increased concentrations of circulating corticosterone in response to stressful events, with transmission of stressed-altered maternal microbiota have long-term effects on gene expression at level of the hypothalamic paraventricular nucleus. The microbiota could regulate the serotonin system in the brain, influencing the hypotalamic–pituitary–andrenal axis and modifying gene expression at hippocampal and hypothalamic levels.	Jašarević et al., 2021 [131] Neufeld et al., 2011 [142]
Anorexia Nervosa	Increased concentration of bacteria of the species *Clostridia*, *Enterobacteriaceae* and *M. Smithii* and reduction in the species *Roseburia*. The intestinal microbiota transforms the proceeds of the diet into a large variety of products including vitamins, amino acid derivatives and short-chain fatty acids, able to modulate the permeability of the blood–brain barrier.	Roubalová et al., 2020 [151] Parker et al., 2020 [167]
Bulimia Nervosa	Increased levels of caseinolytic proteinase B, produced by *E. Coli*, which in turn stimulate autoimmunity response. The activity of the centers of appetite is complexly regulated by an elaborate neuroimmunoendocrine communication system, with the microbiota regulating the activity of adipose tissue and general homeostasis.	Smitka et al., 2021 [154] Lucas et al., 2019 [156]

## Data Availability

Not applicable.
